# Integration of facial features under memory load

**DOI:** 10.1038/s41598-018-37596-2

**Published:** 2019-01-29

**Authors:** K. Ölander, I. Muukkonen, T. P. Saarela, V. R. Salmela

**Affiliations:** Department of Psychology and Logopedics, Faculty of Medicine, University of Helsinki, Helsinki, Finland

## Abstract

Simple visual items and complex real-world objects are stored into visual working memory as a collection of independent features, not as whole or integrated objects. Storing faces into memory might differ, however, since previous studies have reported perceptual and memory advantage for whole faces compared to other objects. We investigated whether facial features can be integrated in a statistically optimal fashion and whether memory maintenance disrupts this integration. The observers adjusted a probe – either a whole face or isolated features (eyes or mouth region) – to match the identity of a target while viewing both stimuli simultaneously or after a 1.5 second retention period. Precision was better for the whole face compared to the isolated features. Perceptual precision was higher than memory precision, as expected, and memory precision further declined as the number of memorized items was increased from one to four. Interestingly, the whole-face precision was better predicted by models assuming injection of memory noise followed by integration of features than by models assuming integration of features followed by the memory noise. The results suggest equally weighted or optimal integration of facial features and indicate that feature information is preserved in visual working memory while remembering faces.

## Introduction

Faces contain socially important information and consequently our visual system is very sensitive locating and detecting faces^1^ and a large network of brain areas is specialized in processing of faces^2,3^. Facial information can be roughly divided into two categories: changeable features, such as emotional expressions, and invariant features, such as identity. Previous studies suggest that changeable and invariant features are, at least partly, processed by different mechanisms^4^. Monkey single cell studies^5^ and human fMRI studies^6^ have shown evidence for norm-based coding of identities, suggesting that identities are represented in a multidimensional facial feature space^7^. Different facial features, however, contribute differently for face perception; the regions around mouth and eyes are most informative^8,9^ and the discriminability of head shape and hair-line is better than discriminability of mouth, eyes and eyebrows^10^.

A common notion in the literature of face perception is that of holistic or configural processing^11–14^, which suggests that the perception of a whole, upright face is different from its parts or faces presented upside-down. The whole face benefit might be due to an optimal integration of facial features. Previously, statistically optimal integration has been studied in face recognition by measuring contrast thresholds^15,16^. In these studies contrast thresholds for identity recognition were measured using facial features in small circular apertures, and compared to the contrast threshold of the whole face (i.e., all features presented at the same time). The results suggested that facial feature integration is optimal^15,16^ or supra-optimal when spatial uncertainty to feature locations was added^17^. Optimal integration of facial form and motion cues was also found in identity matching task with high contrast synthetic faces^18^.

The representations of faces, and visual objects in general, can deteriorate due to several factors. Recognition may be impaired due to visual clutter or noise, holding visual representations in memory for prolonged time, or trying to remember several objects at the same time. According to current understanding of visual working memory, there is a trade-off between the memory capacity and precision^19–23^, that is, the more objects we try to remember, the less precise the memory representations are. For primary visual features, the decline in memory precision due to multiple items in memory can be explained by an increasing noise during memory maintenance. For complex items containing multiple features, the memory noise can have several effects. If the individual features are bound together to form object representations^24^, the memory noise could also affect the binding or integration, in addition to the features. Previous studies suggest independent storing of different features of simple^25–27^ and complex visual objects^28^. Upright faces, however, are remembered better than other complex visual objects^29^ or faces presented upside-down^30^. Thus, for images of human faces, memory noise could corrupt the whole, integrated face representation, it could have different effects on different features if they are independently stored, or it could disrupt the integration or binding as such.

**Figure 1:**
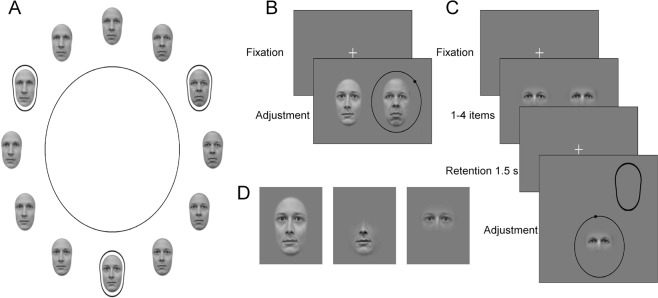
Stimuli and experimental setup. (**A**) Circular identity spaces were created by morphing three original identities with neutral expression to each other (originals marked with a black outline). (**B**) In perceptual matching experiment the observers matched to stimuli presented side-by-side (a black dot moved around the black circle for visual feedback). (**C**) In the memory experiment, 1–4 stimuli were presented and observers adjusted the identity of a probe item to match the identity of a cued item (oval outline). (**D**) All the experiments were conducted with whole faces, isolated mouth (lower part of the face) and isolated eyes (upper part of the face). The identities in the figure are examples, not the ones that were used in the actual experiment.

We studied integration of facial features using high-contrast images of real faces and a task where the observer adjusted the identity of a probe face or an isolated feature to match a target face/feature (Fig. 1A,D). The stimuli were presented side-by-side (perception; Fig. 1B) or sequentially with a retention period in between the target and probe (memory; Fig. 1C). In the memory condition we varied memory load and the observers had to memorize one to four identities. Precision of adjustment was estimated by fitting a wrapped Cauchy distribution to the distribution of adjustment errors. We computed predictions for the whole-face stimulus in the perception condition assuming a model observer who optimally integrates the two features (eye and mouth region). Human observers’ performance was well predicted by this model. We then predicted the performance for the whole face stimulus in the memory conditions assuming that the whole face is affected by memory noise in the same way the individual features are. We tested several models, assuming that either faces are stored as integrated objects or that features are stored separately, and assuming that either most reliable cue is used, cues are equally weighted or optimally integrated. The human data was best predicted by models assuming optimal or equally weighted integration of separately stored features.

## Results

### Precision of adjustments

**Figure 2:**
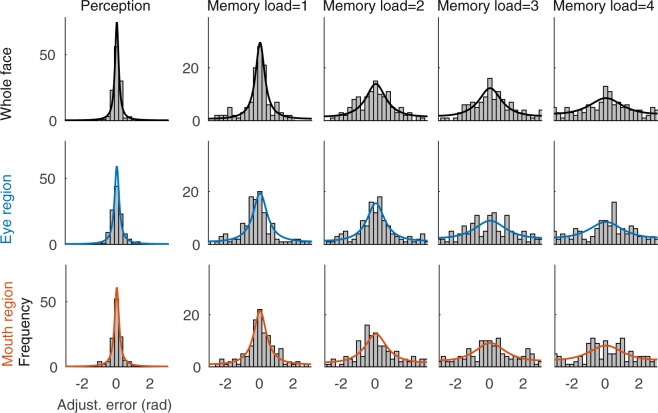
Error distributions of an individual observer (observer 6). The adjustment error distributions (gray bars) and fitted wrapped Cauchy distributions, appropriately scaled (black, blue, and red lines for whole face, eye region and mouth region, respectively).

**Figure 3:**
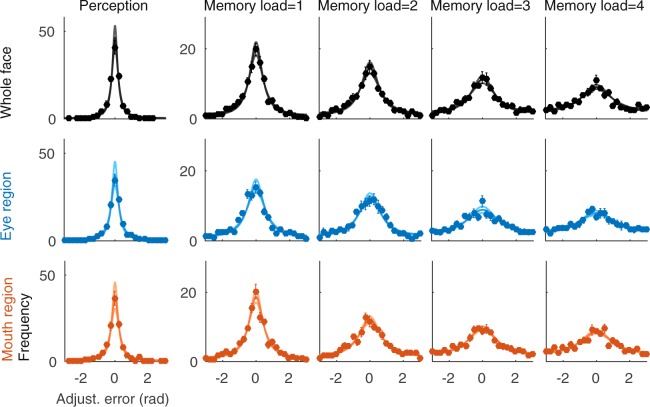
Error distributions averaged over all observers. The average error distributions (circles) and average fitted wrapped Cauchy distributions, appropriately scaled (black, blue, and red lines for whole face, eye region and mouth region, respectively). Error bars depict standard error of mean.

In the perception experiment, the observers viewed two stimuli presented side-by-side and adjusted the stimuli on the right side to match in identity the stimuli on the left side (Fig. 1B). Although the stimuli were constantly visible, the perceptual matching of identity was not perfect. Instead, the adjustment errors formed distributions, which were well fit by a wrapped Cauchy distribution for each individual observer (data and fits for observer 6 shown in Fig. 2, perception data in the first column). The error distributions were narrower for the whole face than for the eyes and mouth stimuli (Fig. 2, first, second and third rows, respectively). The same pattern of results was found in the average data (Fig. 3, first column).

The mean ± *s*.*e*.*m* circular standard deviation of perceptual errors was 0.44 ± 0.049, 0.56 ± 0.084, and 0.56 ± 0.082 radians for faces, eyes, and mouth, respectively. The width of the error distribution was narrower for whole faces than for eyes (one-sided *t*(7) = 2.67, *p* = 0.016; BF_10_ = 5.17) and mouth (one-sided *t*(7) = 3.13, *p* = 0.008; BF_10_ = 8.64), while no difference between the eyes and mouth was found (*t*(7) = 0.035, *p* = 0.973; BF_10_ = 0.336). The corresponding mean ± *s*.*e*.*m* concentration *ρ* values of the fitted wrapped Cauchy distribution, were 0.81 ± 0.022, 0.77 ± 0.037 and 0.77 ± 0.041, for faces, eyes and mouth, respectively. The concentration parameter was higher for whole faces than for eyes (one-sided *t*(7) = 2.20, *p* = 0.032; BF_10_ = 3.020) and mouth (one-sided *t*(7) = 1.96, *p* = 0.046; BF_10_ = 2.288), but no difference was found for isolated eyes and mouth (*t*(7) = 0.070, *p* = 0.946; BF_10_ = 0.337). The Bayesian tests provided confirmatory evidence in favor for our hypotheses (all BFs > 2.8), and in favor for *null hypotheses* when isolated features were compared (BFs < 0.34). Thus the perceptual precision was better for the whole face than isolated features, but the precision for features did not differ from each other.

To further test the difference between the features and faces, two additional fits of Cauchy distributions were made. The first model – reported above – contained separate parameters for all stimulus types. The second model contained different parameters for features and faces, and the third model contained same parameter for all stimulus types. Better fits were obtained with models one and two than model three. There was some individual variability, but in comparison to model three, the AIC scores were smaller for models one and two, mean ± *s*.*e*.*m* were 0.39 ± 1.32 (sum over observers 3.12) and 1.21 ± 1.50 (sum over observers 9.65), respectively. Since the precision for eyes and mouths was so similar, separate parameters for the different features (model one) did not provide additional benefit (AIC difference to model two was −0.82 ± 0.53, sum over observers −6.52).

In the second experiment a 1.5 second memory period was added between the target and probe stimuli, and the observers’ task was to adjust the probe to match the target identity in memory (Fig. 1C). Consequently, the width of the adjustment error distribution increased. This was evident already with just one stimulus to be remembered (Fig. 2/3, second column). Adding the retention interval to the task doubled the averaged standard deviation of errors to 0.92 ± 0.059, 1.09 ± 0.073, and 0.97 ± 0.048 radians for faces, eyes and mouth, respectively. When the memory load was increased from one to four (Fig. 2/3, columns 2–5), the width of the error distributions further increased and the mean ± *s*.*e*.*m* standard deviation of errors for four items was 1.56 ± 0.078, 1.62 ± 0.055, 1.60 ± 0.062 radians for faces, eyes and mouth, respectively). However, even with the largest memory load, the observers were able to memorize the stimuli and the error distributions were not flat (Fig. 2/3, column five).

The effect of memory load on the standard deviation of errors was statistically highly significant (*F*(3,21) = 79.95, *p* < 0.001; Log(BF_10_) = 54.73) as well as the main effect of stimulus type (*F*(2,14) = 7.83 *p* = 0.005; BF_10_ = 44.77). There was, however, no interaction between the stimulus type and memory load (*F*(6,42) = 1.32, *p* = 0.272; BF_10_ = 0.269) suggesting that the memory precision declined similarly for individual features and the whole face. Similar results were found for the concentration parameter of the fitted distributions, main effects of load (*F*(3,21) = 97.95, *p* < 0.001; Log(BF_10_) = 60.470) and stimulus type (*F*(2,14) = 10.637, *p* = 0.002; Log(BF_10_) = 6.411), but no interaction (*F*(6,42) = 0.887, *p* = 0.513; BF_10_ = 0.171). The low BF in the last (0.171) test provides quite strong evidence against the interaction of load and stimulus type.

When fitting additional models to the memory data, better fits were obtained with models that contained a separate concentration parameter for features and whole face, except for highest memory load (mean ± *s*.*e*.*m* AIC differences between third and second model were 0.27 ± 0.94, 1.48 ± 1.48, 0.08 ± 0.63, and −1.27 ± 0.38 (sum over observers: 2.16, 11.86, 0.68, and −10.13); AIC differences between third and first model were 0.03 ± 1.38, 0.41 ± 1.53, −0.21 ± 1.10, and −1.61 ± 0.96 (sum over observers: 0.24, 3.3, −1.64, and −12.85)). And again due to similar concentration for eyes and mouth, separate parameters for eyes and mouth did not provide benefit (AIC difference between second and first model were −0.24 ± 0.96, −1.07 ± 0.31, −0.29 ± 0.69, and −0.34 ± 0.81 (sum over observers: −1.92, −8.55, −2.32, and −2.72)).

One possible explanation for the difference between the features and the whole faces is that the observers enjoyed the whole face condition more and therefore spent more time in adjusting the probe in the whole face condition. However, the Bayesian test revealed evidence *against* an effect of condition (eyes vs. mouth vs. face) on adjustment durations in perception (F(2,14) = 0.752, p = 0.489; BF_10_ = 0.381) or in memory experiment (F(2,14) = 2.126, p = 0.156; BF_10_ = 0.313). In the memory experiment, memory load had no main effect on durations (F(3,21) = 0.559, p = 0.648; BF_10_ = 0.095), and no interaction with stimulus type (F(6,42) = 0.315, p = 0.925; BF_10_ = 0.094). In the latter tests, the evidence against any effects is particularly strong (BF less than 0.1), indicating that observers in all condition made similar effort in adjusting the probe stimulus. In perception experiment, the mean ± *s*.*e*.*m* durations for faces, eyes, and mouths were 11.0 ± 0.81, 10.3 ± 1.32, and 11.7 ± 1.51 seconds, respectively. In memory experiments, observers spent much less time adjusting the probe, the mean ± *s*.*e*.*m* durations for faces, eyes, and mouths were 6.3 ± 0.68, 6.6 ± 0.72, and 6.2 ± 0.73 seconds.

**Figure 4:**
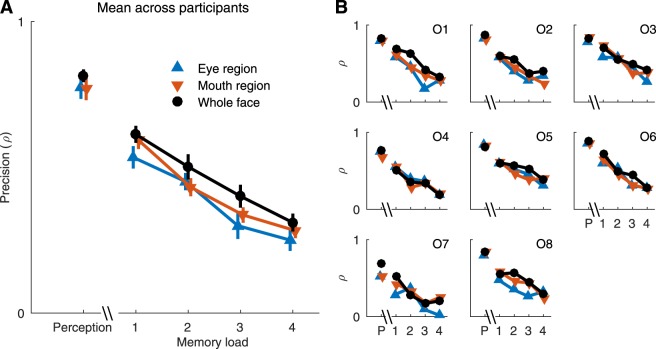
Results from both experiments. Measured precision as the concentration parameter of fitted wrapped Cauchy distributions. (**A**) The mean ± s.e.m, across all observers. (**B**) The precision of all observers. Color conventions as in Fig. 2. P = Perception.

To summarize all the results, the average and individual precision (i.e., concentration parameter *ρ* of the fitted wrapped Cauchy distributions) in perception and memory experiments are shown in Fig. 4A,B, respectively. Perceptual precision was substantially better than memory precision, as expected. On average, precision was better for the whole face than for the isolated features in every condition (Fig. 4A), although there was some individual variation (Fig. 4B). For 7/8 observers the perceptual precision and for 5–7/8 observers the mnemonic precision was better for the whole face than for isolated features, depending on the memory load (Fig. 4B).

### Modeling

**Figure 5:**
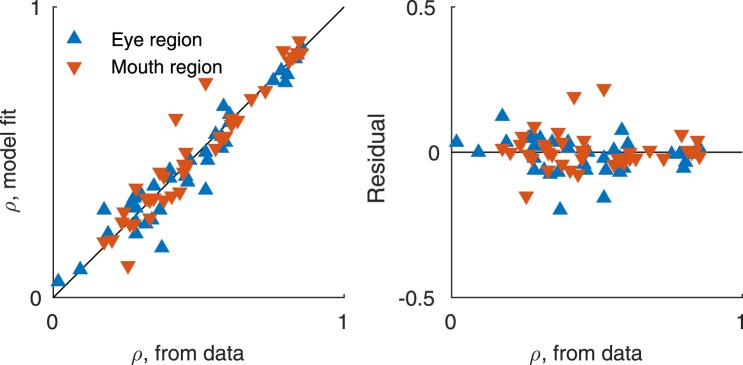
Fit and measured precision values, all observers and memory loads. See text for details.

Since the precision of the isolated features did not reach the precision of the whole face (Fig. 4A), it seems that the facial features are integrated when matching facial identities of the whole face. To quantify the feature integration, we predicted the whole face performance based on the performance with isolated features and assuming optimal integration. We assumed that three independent sources of noise (feature, memory delay, and load) limit precision. We estimated the precision of each type of noise from the isolated feature data (eyes and mouth), separately for each observer (see Methods). The fits captured the effects of memory on precision well, and the estimated precision in each condition and for every observer was very close to the observed data (Fig. 5).

**Figure 6:**
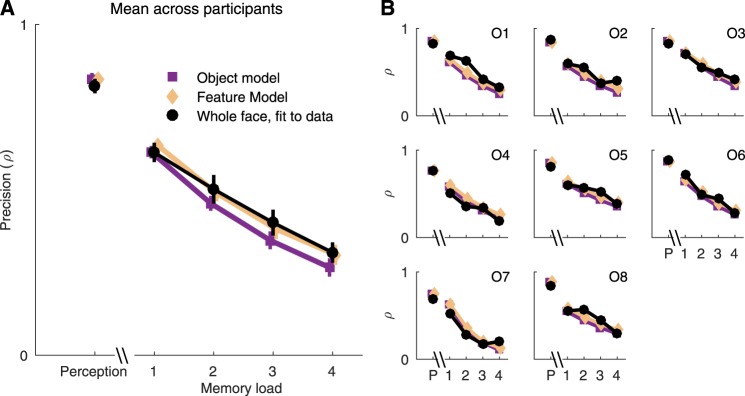
Predicted and measured precision values for the whole face. (**A**) The average precision across all observers. Feature Model predicted the measured whole face precision most accurately. (**B**) Precision values of each observer. The accuracy of both models varied across observers, overall Feature Model predicted the precision better than the Object Model. P = Perception.

We then tested for integration of features in the whole-face condition. We devised two versions of an optimal-integration model, in which memory noise corrupts perceptual precision before (*Feature Model*) or after (*Object Model*) the integration of features, and compared human observer performance against model predictions. In addition, we fitted two versions of an equal-weighting-of-cues model, in which the cues were integrated before or after memory noise, and a most-reliable-cue model. The predictions of the optimal-integration models are shown in Fig. 6, along with the whole-face data replotted from Fig. 4. The perception-only (no memory load) prediction is identical for the two models, and especially the average data (Fig. 6A) is in accordance with this prediction. Both models predict a similar and systematic decline in performance as a function of memory load, but the Feature Model, in which memory noise corrupts precision *before* integration, predicts better performance overall in the memory conditions. Averaged over observers, the Feature Model also more accurately predicts performance. The individual observer data (Fig. 6B) are noisy, but based on likelihood ratios, the Feature Model better predicted performance for 6 out of 8 observers. When comparing all fitted models, for 6/8 observers, higher likelihoods were found for models containing integration of features after memory noise rather than before memory noise. For 3/8 observers the best model contained optimal integration, for 3/8 observers the best model was equal weighting, and for two observers the most reliable cue provided the best fit for data. The predictions of the optimal-integration and equal-weighting-of-cues models were very similar.

## Discussion

We investigated integration of facial features in a face identity matching task, and tested whether adding a retention period and an increasing memory load disrupts feature integration in memory. To this end, we measured adjustment errors for facial features and the whole face while observers matched stimuli presented side-by-side as well as stimuli separated by a memory interval. The perceptual and mnemonic precision for the whole face was better than for the isolated features, and the precision decreased due to retention period and memory load. Memory precision for the whole face was well predicted by models with late integration of noisy features. Alternative models – early integration of features followed by an additive memory noise – underestimated memory precision. We compared optimal integration with equal weighting of cues and choosing most reliable cue. The results suggest that facial features are integrated in statistically optimal fashion or by equally weighting features, and that feature information is preserved while memorizing complex objects.

A growing amount of evidence suggests flexible resources in visual working memory^31,32^. According to current understanding, we can trade precision with capacity, that is, remember only few items very precisely or larger amount of objects with less resolution. This can be achieved either by continuous resources^21,33^, variable precision^19,20^ or by averaging discrete memory representations for improved precision^23^. Most studies on the precision of working memory have used simple visual features or shapes as stimuli to be memorized. With more complex or naturalistic stimuli, such as images of human faces, the precision of memory representations can decrease in several ways. We can remember complex objects either as a whole object, or as a collection of features combined with binding information^24,34^.

Memory binding is typically studied with combining simple visual features, such as color and orientation. Previous studies suggests that memory loads affects binding^25^ and emphasize feature locations, that is, features are bound mainly due to the shared location^35,36^. Features of simple visual objects are independently stored^25–27^. Similarly, complex, real-world objects are not remembered as unitary or bound objects since the memorability of features are partly independent^28^. Our results with faces suggest that memory noise similarly degrades both the isolated features as well as the integrated objects, that is, there was no interaction between the memory load and stimulus type. Furthermore, predicting the memory precision for the whole face with different models favored the models in which memory noise was added before feature integration. This suggests that unfamiliar faces are stored in memory as collection of features or that feature information is preserved while storing facial identity in working memory. Interestingly, storing a face as an integrated object is not the most optimal strategy since – according to our modeling – storing features separately predicted better memory performance. Although there was some variability, majority of our observers seemed to utilize this strategy.

It has been suggested that visually complex objects employ more working memory resources than visually simple objects^37^. In our experiment the whole-face stimuli were remembered more precisely than the single-feature stimuli, although faces are visually more complex. However, the precision declined similarly for both whole face and isolated features. There are several advantages for faces compared to other visual objects. Faces are remembered better than other objects, if the encoding time is long enough^29^, and more precisely than line orientations when multiple items need to be remembered^38^. Face inversion effect has also been reported during memory maintenance, that is, memory precision for upright faces is better than for upside-down faces^30^. Our results suggest that the memory advantage for faces is not due to storing faces as integrated objects.

The difference in precision between facial features and the whole faces was not as large as we expected on the basis of holistic or efficient processing of whole upright faces. Furthermore, the precision for mouth and eyes was quite similar in all conditions. These could be due to our feature stimuli; the whole face was split in half and we compared identity information in the lower and upper part of the face. The whole face benefit, as well as the difference between the features, would likely be larger if facial features would be chosen with a smaller aperture, i.e., just a left eye, or an eye without the eyebrow. Furthermore, the reliability of different cues could be varied with varying aperture size, which in turn would make predictions of optimal integration and equal weighting of cues more different. In the current data, we cannot separate these two models.

All the identities we used were unfamiliar to the observers. Identity processing differs between unfamiliar and familiar faces, and facial expertise is more pronounced with familiar faces^39^. With familiar faces, observers would have had much more exposure to the stimuli and different memory representations, and they would likely have used different strategies or weighting of features while memorizing identities. However, as the main interest of the study was short-term or working memory, long-term memory representation of familiar faces could have affected these processes, as observers could have used other identity information, such as names, while memorizing faces.

Previously optimal integration of facial features has been shown for detection of faces at contrast threshold^15–17^ as well as discrimination identity of dynamic synthetic faces^18^. In accordance with these studies, our results suggest optimal integration or equal weighting of facial features in an adjustment task. In comparison to the previous integration studies, we added a retention period and varied the memory load. The memory maintenance or load did not have much effect on the integration as such. In conclusion, our results suggest optimal integration or equal weighting of facial features in identity matching task, and that faces are stored as collection of features, which are integrated during retrieval.

## Methods

### Observers

Eight observers (4 female, 22–31 years) with normal or corrected to normal vision and without known deficits in face perception participated in the experiment. All observers received a study credit for participating. All the experiments were conducted in accordance with the Declaration of Helsinki. A written informed consent was collected from the observers before the measurements, and the experiments were approved by the Ethics Review Board in the Humanities and Social and Behavioural Sciences of the University of Helsinki.

### Stimuli

We chose 60 face images depicting different identities from the Radboud^40^ and FACES^41^ databases. All faces had a neutral expression. Half of the identities were female and half male. Images were divided into groups of three and morphed from one identity to another by using Abrosoft FantaMorph software. As a result, 20 different circular identity spaces, which each contained 300 images in total, were formed. In each of these spaces the identity changed continuously between the three original identities (Fig. 1A). The gender did not change within the identity space.

The three different stimulus types, whole face, mouth region (lower part of the face) and eye region (upper part of the face), were obtained by applying Gaussian masks to the images (Fig. 1D). The masks were identical across all identities. The whole faces were first extracted from the original images with a mask, which shape was determined by two radial frequency components (first component: RF = 2, amplitude = 0.22, phase = 270°; second component: RF = 3, amplitude = 0.04, phase = 180°). The eye and mouth regions were extracted from the whole face with masks defined by a sum of Gaussians, i.e., the eye region was extracted with two Gaussians around the left and right eye and the mouth region with two Gaussians around the nose and mouth. The standard deviations of the Gaussian masks for the left and right eye were ${\sigma }_{x}=1.6^\circ$ and ${\sigma }_{y}=0.8^\circ$, for nose ${\sigma }_{x}=0.6^\circ$ and ${\sigma }_{y}=0.8^\circ$, and mouth ${\sigma }_{x}=2.0^\circ$ and ${\sigma }_{y}=1.2^\circ$. The size (width/height) of the whole face, eyes and mouth were 4.1° × 6.0°, 4.1° × 1.7° and 3.7° × 3.2°, respectively. Gray-scale faces/features were displayed on a mid-gray background and the RMS-contrast of the stimuli was 0.19 ± 0.01. All the image processing was conducted with Matlab.

Experiments were conducted in a dimly lit room. Stimuli were shown on a linearized VIEWPixx monitor (VPixx Technologies Inc., Canada). Observers sat 92 cm away from the monitor and their head rested on a chin-forehead stand. The viewing area extended 29.5 × 18.8 degrees. The stimulus presentation was controlled with Psychophysics Toolbox extension of Matlab^42^.

### Procedure

The precision of perceiving and remembering facial identity was measured with the method of adjustment. Two experiments (perception and memory) were conducted using three different types of stimuli: (1) the whole face, (2) mouth region only, and (3) eye region only. In the perception experiment, a target stimulus and a probe stimulus were presented simultaneously, side-by-side (Fig. 1B). The observers’ task was to adjust the identity of the probe to match the target. The target and the probe were always similar, i.e., whole face, or eye or mouth region. The target was always on the left side of the display and the probe on the right side of the display. The observer used up/down arrow keys for coarse adjustment (4.8° steps in the identity space) and left/right arrow keys for precise adjustment (1.2° steps in the identity space). When the observer was content with the adjustment, he/she initiated the next trial by pressing the spacebar. The maximal adjustment time was limited to 30 seconds. The positions of the target and the starting position of the probe stimulus in the identity space were random, except the probe was initially at least ± 30° away from the target. For visual feedback on the identity space, there was a thin black circle around the probe stimulus, and a black dot moved along the circle according to observers’ adjustment (Fig. 1B).

In the memory experiment, memory precision was measured while varying the memory load. First, 1–4 stimuli were shown and the observers’ task was to memorize the identities of the stimuli. The stimuli were always shown on the same, fixed locations, and for 0.5 s per face, i.e., one face was shown for 0.5 s and three faces were shown for 1.5 s. After a 1.5 s retention period, a probe stimulus was presented on the bottom of the screen, and the observers’ adjusted the probe stimulus to match the target identity, which was indicated with a spatial cue (outline of the face; Fig. 1C). The adjustments were done similarly as in the perception experiment. In the conditions with more than one stimulus, all of the stimuli were always from different identity circles.

All observers conducted the perception experiment first. For each experiment and condition, 120 trials were measured in two blocks of 60 trials. The order of the four memory loads and three stimulus types, and the two blocks in the memory experiment were randomized and balanced across observers. In every block, all of the 20 identity spaces were probed three times, once in each third of the circular space.

### Data analysis

The adjustment error on each trial was obtained by computing the angle between the adjusted identity and the target identity in the circular identity space. To quantify the precision of the adjustments, we fit wrapped Cauchy distributions^43^ to the adjustment error distributions. Wrapped Cauchy density function is given by:

1 $$f(\theta ;\mu ,\rho )=\frac{1}{2\pi }\frac{1-{\rho }^{2}}{1+{\rho }^{2}-2\rho \,\cos (\theta -\mu )}$$

where θ is the angle, µ is a location parameter, and the concentration parameter ρ defines the precision, varying between 0 (uniform circular distribution) and 1 (distribution concentrated at µ). Fitting was done separately for each observer, condition, and stimulus type by numerically finding the maximum likelihood values for the parameters given the data. The mean on the distribution (location parameter µ) was set to zero. We used wrapped Cauchy instead of von Mises distribution because von Mises failed to capture the shape of the error distribution, especially when the error distribution was very concentrated. We quantified the difference in the goodness of fit between the wrapped Cauchy and von Mises by comparing their log-likelihoods. Average log-likelihood ratio for wrapped Cauchy vs. von Mises was 21.97 ± 7.97, confirming that wrapped Cauchy gave a better fit. In addition, we confirmed that the shape of the distributions did not differ between eyes, mouths and whole faces by fitting wrapped stable distribution^44^ to the data with shape and concentration as free parameters. The shape parameter value 1 corresponds to a wrapped Cauchy distribution and value 2 corresponds to a wrapped Gaussian distribution. The mean ± *s*.*e*.*m* shape values were 1.08 ± 0.048, 1.14 ± 0.066 and 1.07 ± 0.091 for mouth, eyes, and face, respectively, and did no differ significantly from each other. All further analyses and modeling were done using wrapped Cauchy.

In contrast to some previous studies on visual memory, we used only a circular distribution, not a mixture of a circular and a uniform distribution, since the wrapped Cauchy alone gave a good fit for the data. Further, for the modeling (see below) it was essential to get a good estimate of precision in each experimental condition, and this becomes problematic with a mixture of a circular and a uniform distribution, since the spread of the observations could be absorbed either by the weight of the uniform distribution or the concentration parameter of the circular distribution, especially when the spread is large.

Statistical analyses were conducted with JASP software^45,46^. Paired sample t-tests and repeated measures ANOVAs were used as well as Bayesian paired samples t-tests and Bayesian ANOVAs. The t-tests were two-sided unless otherwise noted. The Bayes Factors (BF) are reported relative to the null model and in two-way ANOVAs the BF are reported relative to the null model including the other effects.

### Modeling

We developed a model to investigate whether facial features are optimally integrated for identification. The model assumes independent processing and statistically optimal integration of noisy features, and a further corruption of precision during memory maintenance by additional neural noise.

During the experiment, the observer adjusts one of the two stimuli so that the ‘perceptual distance’ between the two is minimized. The model observer does this using a noisy decision variable *r*, which is a difference of noisy ‘internal responses’ to the two stimuli: *r = r*_1_ *− r*_2_. We assumed there are three independent sources of noise in the task. First, there is noise related to the coding of the features, which corrupts the responses to both the target and the probe stimuli. Second, we assumed two sources of zero-mean noise related to memory: noise related to the delay or retention period itself, and noise that increases with memory load. Convolution of two wrapped Cauchy distributions is again a wrapped Cauchy distribution with a concentration parameter that is the product of the concentration parameters of the original distributions^47^. The effect of memory noise can thus be modeled as:

2 $$\rho ={\rho }_{feature}^{2}{\rho }_{delay}{\rho }_{load}^{n}$$

where ρ_feature_ reflects the precision in the representation of the eye or mouth region (ρ_eyes_ or ρ_mouth_, squared because on each trial there are two stimuli, the target and the probe), ρ_delay_ is the precision of the noise due to the retention period, and ρ_load_ is for the noise due to each of the *n* additional items kept in memory. In the perception condition, ρ_delay_ and ρ_load_ are both equal to 1. We fit this model to the precision estimates extracted from the data from isolated eyes and mouth conditions to estimate the noise related to the two features, memory delay, and memory load, separately for each observer. We set the mean to zero (location parameter µ = 0) for all distributions.

We then predicted the observer’s performance in the whole face task assuming optimal integration of the features (i.e, maximum likelihood estimation of identity given responses to features). As we did not have ‘cue-conflict’ conditions, that is, the eye and mouth region always had the same identity, we assume the responses to the two features have the same mean. To model the perception condition, we took the precision estimates for the features ρ_eyes_ and ρ_mouth_ and simulated 10^4^ ‘trials’ by drawing random samples (‘responses’) from the corresponding wrapped Cauchy distributions. For each response, we computed the likelihood function for the true stimulus value given the response. As we assumed independent processing of the features, the combined likelihood is simply the product of the feature-specific likelihoods:

3 $$p({r}_{eyes},{r}_{mouth}|I)=p({r}_{eyes}|I)p({r}_{mouth}|I)$$

where *I* is the stimulus value (identity). The model observer made a maximum likelihood estimate of the identity by picking the value of *I* that maximized this likelihood. To quantify the model observer’s performance, we fit a wrapped Cauchy distribution to the errors in the same way as we did for the human data.

To model the memory conditions, we devised two versions of the optimal integration model, which differ in how memory noise corrupts the responses: In the first model, the features are first integrated, and the maximum likelihood estimate is then corrupted by memory noise. In the second model, the responses to the features are first corrupted by memory noise before being integrated. Additionally, we tested a model with equal weighting of the two cues (as we did not systematically manipulate cue reliability, this is likely to be very close to the optimal-integration model), and a model where the observer chooses the most reliable cue.

## Data Availability

The data is available in Open Science Framework repository (osf.io/v79h6/).
